# Enrollment and associated factors of the national health insurance program of Nepal: Further analysis of the Nepal Demographic and Health Survey 2022

**DOI:** 10.1371/journal.pone.0310324

**Published:** 2024-10-03

**Authors:** Devaraj Acharya, Sushil Sharma, Kristin Bietsch

**Affiliations:** 1 Research Centre for Educational Innovation and Development [CERID], Tribhuvan University, Kathmandu, Nepal; 2 Prithvi Narayan Campus, Tribhuvan University, Pokhara, Nepal; 3 Avenir Health, Glastonbury, CT, United States of America; Global Center for research and Development (GCRD), UNITED STATES OF AMERICA

## Abstract

The focus of this study was on the current enrollment status of the government-funded health insurance (HI) program in Nepal, which is necessary to achieve universal health coverage by 2030. Despite the government’s commitment, the program faces challenges of low enrollment and high dropout rates, hindering progress towards this goal. With a purpose to find out the associated factors for enrollment in HI, the cross-sectional study employs secondary data obtained from the Nepal Demographic and Health Survey 2022. A multi-stage sampling method yielded a representative sample of 14,280 households, and an interview was conducted with 14,845 females and 4,913 males aged 15–49. A weighted sample was employed and subsequently analyzed through the use of R. The analysis reveals a concerningly low enrollment rate, with only 10% of the surveyed population possessing government HI. Furthermore, significant geographical disparities were found to exist—Koshi Province had the highest coverage (21.8% men and 20.4% women), while Madhesh Province lagging far behind (3.1% men and 2.7% women). Additionally, the enrollment rates correlated positively with urban residence, higher socioeconomic statuses, and employment, with no subgroup surpassing 30% coverage, though. The study demonstrates a positive association between HI and healthcare utilization, with insured individuals exhibiting a higher likelihood of visiting health facilities and reporting fewer access-related issues. Respondents with higher levels of education and greater wealth were significantly more likely to enroll in HI than those with basic education and middle-level wealth, respectively. This pattern holds consistently for both males and females. These findings suggest that the program, aiming for 60% coverage by 2023/24, is currently off-track. Policymakers should interpret these data as a call for action, prompting the development and implementation of the targeted interventions to address enrollment disparities across Nepal. By focusing on the low-coverage areas and the vulnerable populations, the program can be strengthened and contribute meaningfully to achieving universal health coverage by 2030.

## 1 Introduction

With an aim to increase the citizens’ access to quality healthcare without much financial hardship, the Government of Nepal (GoN) initiated a comprehensive public health insurance program in 2016. The Health Insurance Board (later named the Social Health Security Development Committee before the enactment of the Health Insurance Act, 2017 and the Health Insurance Regulations, 2019 [1, 2]), was established to implement, regulate, and monitor the Health Insurance Program (HIP). The HIP was initially implemented in three districts and has now been expanded to all 77 districts, covering all local authorities [3] that aims to increase accessibility, affordability, and availability (3As) of quality healthcare services [4]. It has also been noticed that health insurance promotes 3As of healthcare services [5, 6].

The Constitution of Nepal (CoN) recognizes access to essential health services as a fundamental right of all citizens, adhering to the principles that ensuring access to quality healthcare regardless of their ability to pay is the responsibility of the state, that the state has the responsibility to ensure basic health services at no cost, that all citizens have equal access to healthcare, and that no one shall be deprived of emergency healthcare [7]. The GoN also advances the right to receive emergency health services, thereby leaving no one to be denied the treatment of a life-threatening illness or injury [7]. However, the data show that the out-of-pocket expenditures for healthcare are higher in Nepal (51.26%) [8] than in the neighboring countries, though Nepal, as a member of the World Health Organization (WHO) and the United Nations (UN), the GoN holds a significant responsibility towards this issue, entailing the sustainability of healthcare financing to achieve the ambitious targets outlined in the third Sustainable Development Goal (SDG)–ensuring good health and well-being, hence guaranteeing access to quality, essential healthcare services for all individuals, irrespective of their income or social standings [9, 10].

The 15^th^ national periodic plan (fiscal year 2019/20–2023/24) states that the GoN has a strategy to maintain a sustainable healthcare financing mechanism by increasing investments in health. The strategy includes an integrated health financing strategy and quality healthcare services through health insurance, which was expected to cover 60% of the total population by the year 2023/24, which was 7% in 2018/19 [11]. Contrarily, the GoN has only allotted two to three percent of the overall national budget [12] or about one percent of the total gross domestic product for the health sector, an amount considered by experts as inadequate [13, 14] to achieve the ends mentioned above as well as the international commitments it has made [15]. This paucity of budget has negatively affected the implementation of the national health insurance program, which is expected to improve sustainable healthcare [11] though good governance, political stability, and demographic balance are equally important for ensuring enrollment in health insurance [16].

As per the provision of the Health Insurance Board (HIB), households with up to five members pay NPR 3500 (US$ 26.38), with each additional member contributing an additional NPR 700 (US$ 5.28). A five-member family will have health service coverage up to NPR 100,000 (US$ 753.58), and each additional member can obtain additional services amounting to NPR 20,000 (US$ 150.72) per individual with a maximum ceiling of NPR 200,000 (US$ 1,507.16).^2^ Patients with critical illnesses (cancer, heart disease, kidney disease, head injuries, spinal injuries, sickle cell anemia, Parkinson’s disease, and Alzheimer’s diseases) [2] will receive an additional coverage of NPR 100,000 (US$ 753.58). According to the Nepal Demographic and Health Survey (NDHS) 2022, approximately 12% of women age 15–49 and 13.3% of men age 15–49 were enrolled in a HIP [17].

A study from Baglung and Kailali districts showed that the residents were interested in enrolling in health insurance and willing to pay more than the contribution amount [18]. However, the enrollment rate observed was low, and the dropout rate was high [19]. The NDHS 2022 included health insurance for the first time on its sixth survey comprising 13,786 households (samples) nationwide [17], showing that only about 10 percent of the total population aged 15–49 years were enrolled in the health insurance program.

This paper aims to further explore factors associated with health insurance enrolment deriving from the related descriptive statistics available in the DHS final report. Therefore, this research aims to examine the factors associated with HIP enrollment in Nepal. In addition, we explored the association between health insurance and access to care by examining if the insured were more likely to visit health facilities, and if they were less likely to report the problems of accessing care. It is expected that the study results will provide the policymakers with further information that may be useful for increasing the use of the GoN’s health insurance scheme.

## 2 Materials and methods

The data for this study were obtained from secondary sources provided by the DHS Program. Comprehensive methodological details are available in the NDHS 2022 report [17]. A portion of the data analysis and presentation processes are described in the following sections.

### 2.1 Design

This paper presents a secondary data analysis of the NDHS 2022. Nepal has conducted this nationally representative survey approximately every five years since 1996, although this is the first time that the health insurance questions were included. We extracted the data from households, men’s (age 15–49), and women’s (age 15–49) datasets [17]. The data are available from the DHS program. The health insurance status was asked using the women’s and men’s questionnaires.

### 2.2 Study setting

The NDHS 2022 constitutes a nationally representative survey. As such, the entire households within Nepal were considered the study population. To ensure the coverage, the NDHS employed a multistage sampling strategy encompassing all provinces and both rural and urban settings. Moreover, the NDHS utilized an updated sample frame that consisted of 36020 sub-wards as provided by the National Statistics Office [17].

### 2.3 Study participants

The households were selected with an updated sampling frame developed by the Central Bureau of Statistics. In all households, women, aged 15–49 years, who were permanent residents or visitors who had slept in the household the night before, were interviewed. In half of the households, men, aged 15–49, who were residing permanently or visitors who had slept in the household the night before, were interviewed. A total of 14,845 women and 4,913 men selected from 13,786 households were interviewed using the women’s and men’s questionnaires, respectively [17].

### 2.4 Variables and measures

The participants were asked if they currently were insured and, if yes, what type. Enrollment in the government funded health insurance was considered a dependent variable, while the sociodemographic characteristics of the households, men, and women were considered independent variables. Province, residence, caste, marital status, education, wealth, household size, age, self-rated health, and occupation were included as the socioeconomic variables for the participants. A husband living with his wife was included as an independent variable of each married woman.

We observed the association between possessing insurance and accessing care for women (due to the questionnaire constraints). Moreover, we examined if the women with insurance (independent variable) were more likely to have visited a health facility in the last year and if they expressed the major issues with accessing care (dependent variables). All women were asked if such categories as obtaining permission to go to the doctor, obtaining money for advice or treatment, distance to the facility, and not wanting to go alone where the following were “big problems” in accessing care. We used the weighted data from the DHS datasets.

### 2.5 Data analysis

For the participants, we calculated the percent of insured with insurance for subgroups of each variable of interest. We also calculated the population distribution of those with and without government insurance. All the statistical analyses were performed using R [20]. We used bivariate and multivariate logistic regressions to determine statistically significant differences within the independent variables. For one variable, the husband living with his wife, we restricted our sample to married women. To examine the association between possessing insurance and accessing care, we conducted three regressions per outcome: first, a bivariate regression of insurance on a dependent variable; second, adding controls for travel time to the nearest facility and means of transport to the nearest facility; and third, adding the control variables included in the women’s regression. Before performing the multivariate analysis, we assessed the multicollinearity issue and whether there were serious multicollinearity issues among the variables [21].

### 2.6 Ethical considerations

The survey proposal of the NDHS 2022 was reviewed and approved by the Nepal Health Research Council (Ref. #678: 494/2021 P on September 30, 2021) and the ICF Institutional Review Board (ICF Project # 180657.0.001.NP.DHS.01 on August 25, 2021). The written consent was obtained prior to the interview [17]. This research used anonymized responses in the secondary data analysis. Moreover, an approval letter was obtained from ICF (DHS Program) for a further analysis of the NDHS 2022 datasets.

## 3 Results

This paper is a product of the DHS Program Mentored Papers Program, which aimed to publish research article utilizing DHS datasets. While related findings have been previously published in the DHS working paper series [22], the pertinent results are presented in detail below.

### 3.1 Descriptive statistics

In the 2022 NDHS, only 13.3% of men and 12% of women in Nepal had health insurance, with 10.2% and 10.8% enrolled in the government-implemented health insurance scheme.

As can be seen in Table 1, the level of government-funded health insurance coverage with men (10.2%) and women (10.8%) is similar. For both genders, those living in Koshi Province have the highest level of health insurance, over 20%. Madhesh Province has the lowest, that is 3.0%. Of the men with government insurance, 38.0% live in Koshi, compared to only 15.6% of them without insurance. For the women, the distribution is similar, with 31.7% of them with insurance living in Koshi, contrasted to 15.0% without it. In Madhesh Province, 21.9% of the men and 22.1% of the women have no insurance, contrasted to just 6.2% of the men and 5.0% of the women with it. These disparities may be indicative of the differences in the implementation or the effectiveness of the government insurance programs across those provinces.

**Table 1:** Distribution of government implemented health insurance by socio-demographic characteristics.

Variables	Category	Percent of Population with Government Insurance		Population Distribution: Men		Population Distribution: Women	
		Men (%)	Women (%)	With Government Insurance (%)	Without Government Insurance (%)	With Government Insurance (%)	Without Government Insurance (%)
		10.23	10.81				
Province	Koshi	21.84	20.40	38.33	15.63	31.70	14.99
	Madhesh	3.12	2.68	6.19	21.90	5.03	22.12
	Bagmati	8.37	11.46	20.21	25.22	21.87	20.48
	Gandaki	11.70	16.63	9.01	7.75	14.52	8.82
	Lumbini	9.03	9.39	14.58	16.74	15.75	18.41
	Karnali	12.28	10.33	6.51	5.29	5.85	6.16
	Sudurpashchim	7.31	6.63	5.17	7.46	5.29	9.02
Residence	Urban	10.99	12.19	75.73	69.86	77.32	67.50
	Rural	8.40	7.80	24.27	30.14	22.68	32.50
Religion	Hindu	10.84	11.55	86.79	81.37	89.08	82.66
	Buddhist	6.92	6.02	5.35	8.20	3.64	6.89
	Muslim	5.30	2.71	2.43	4.95	1.15	5.01
	Kirat	11.20	13.46	3.11	2.81	3.07	2.39
	Christian/Other	9.02	10.84	2.32	2.67	3.06	3.05
Caste	Hill Brahmin/Chhetri	17.21	18.13	42.20	23.13	46.93	25.67
	Terai/Madhesi	4.91	5.41	8.96	19.78	7.87	16.67
	Dalit	9.97	7.57	13.06	13.43	10.57	15.64
	Janajatis	8.89	9.79	33.07	38.62	33.14	36.98
	Muslim/Others	5.78	3.49	2.72	5.05	1.50	5.04
Marital Status	In union	10.96	10.80	67.66	62.61	75.25	75.32
	Never In Union	8.99	10.92	31.62	36.48	21.79	21.55
	Formerly In Union	8.29	10.28	0.72	0.91	2.96	3.13
Education	None	2.54	4.49	1.99	8.69	10.62	27.38
	Basic	6.40	7.45	24.17	40.29	21.34	32.12
	Secondary	13.02	15.99	58.12	44.25	57.80	36.79
	Higher	20.94	25.08	15.72	6.76	10.25	3.71
Wealth	Poorest	5.08	4.14	7.60	16.17	6.78	19.03
	Poorer	8.21	7.38	15.24	19.41	13.15	19.99
	Middle	8.21	7.99	15.63	19.92	15.08	21.04
	Richer	10.75	13.05	24.29	22.97	26.01	20.99
	Richest	16.46	19.95	37.24	21.53	38.99	18.95
De Jure Household Size	1 Member	9.48	9.26	2.24	2.43	1.52	1.81
	2 Members	10.46	10.36	7.44	7.25	9.25	9.70
	3 Members	10.60	11.37	18.05	17.35	19.28	18.21
	4 Members	9.73	11.17	23.01	24.32	24.27	23.38
	5 Members	10.52	12.50	20.29	19.66	21.48	18.23
	6 Members	11.78	10.32	15.09	12.88	11.24	11.83
	7+ Members	8.94	8.53	13.88	16.11	12.96	16.83
Age in Years	15–19	6.61	8	12.95	20.85	13.18	18.36
	20–24	11.08	9.55	18.91	17.28	15.70	18.02
	25–29	8.81	11.87	12.56	14.81	18.01	16.21
	30–34	9.92	11.93	12.16	12.58	15.94	14.26
	35–39	14.19	11.07	18.04	12.42	13.97	13.60
	40–44	11.02	12.53	13.25	12.19	12.72	10.76
	45–49	12.29	12.64	12.13	9.86	10.50	8.79
Self-Rated Health Status	Very Good	7.50	5.89	6.31	8.86	3.03	5.87
	Good	10.80	8.85	41.12	38.69	22.99	28.69
	Moderate	10.05	11.94	46.95	47.88	62.68	56.02
	Bad/Very Bad	12.32	12.69	5.63	4.57	11.30	9.42
Occupation	Employed	15.32	21.67	38.66	24.34	31.28	13.71
	Agriculture	9.86	8.45	22.67	23.62	37.56	49.31
	Manual	6.62	8.91	23.89	38.36	6.86	8.50
	Unemployed/Others	10.96	9.37	14.78	13.68	24.30	28.49

Source: Nepal Demographic and Health Survey (NDHS) 2022

In terms of residence, the data showed a notable contrast between the urban and the rural areas in government insurance coverage. The urban areas have higher coverage rates than the rural areas for both genders. In urban settings, women have a 12.2% coverage rate, while men have 11%. In contrast, the women have a coverage rate of 7.8%, while the men have 8.4% in rural areas.

With reference to religion, the Kirat group has the highest insurance coverage, at 11.2% for men and 13.5% for women. However, this group comprises only around 3% of the population. The Hindus have the next highest coverage of 10.8% for the men and 11.6% for the women. All other religious groups have insurance coverage at or below the national average, with the lowest coverage among Muslims, with 5.3% of the men and 2.7% of the women. Regarding caste, the Hill Brahmin and Chhetri have the highest government insurance coverage, with 17.2% of the men and 18.1% of the women, while the Terai/Madhesi and Muslim/others have the lowest coverage of government health insurance.

As regards marital status, there is little variation among the women with the government insurance, which comprises 10.8% of married, 10.9% of never-married women, and 10.3% of formerly married. However, 11% of the men with government health insurance are in a union, followed by 9.0% of them who have never been in a union, and 8.3% of them are formerly married men.

Educational disparities exist in the government insurance coverage for those having no formal education, even at the lowest levels, in contrast to those with higher education who have the highest level of coverage. However, the highest coverage group, women with higher education, is only 25.1%. Wealth follows a similar pattern, with the lowest coverage among the poorest and the highest coverage among the richest residents. The richest quintile includes 37.2% of the men and 39% of the women with insurance. Fewer than 10% of the residents with insurance are in the lowest wealth quintile. Concerning the household size, households with 7 or more members have the lowest rate of government insurance, although there is less variation across the household size than with the other independent variables.

For both men and women, the 15–19 age group was the least likely to have insurance. With the respondent’s self-rated health, those who said they had bad or very bad health (grouped due to the small sample size) have the highest rate of insurance, while those with very good health have the lowest rate. Those who are employed (but not in manual labor or agriculture) had the highest rates of government insurance coverage. The rates were lowest for men who worked in manual labor. Finally, for married women (Table 2), women whose husbands live with them have higher rates of insurance than women whose husbands are staying elsewhere.

**Table 2:** Husband away and enrollment in health insurance.

Husband Away	Distribution of Women enrollment in health insurance (%)	Women having Insurance (%)	Women having no Insurance (%)
Husband living with wife	11.88	72.66	65.28
Husband staying elsewhere	8.70	27.34	34.72

Source: Nepal Demographic and Health Survey (NDHS) 2022

Table 3 presents the data on women’s visits to healthcare facilities in the last year. The data presented under two categories: “Visited” and “Didn’t Visit.” Of the women who visited health facilities, 12% had government-funded health insurance, while 9% of the population who did not visit a facility had it. Overall, most of the women visited a health facility in the last year, with 76% of them, who visited, having insurance, compared to 69% of the women without insurance.

**Table 3:** Distribution of health insurance enrollment among women.

Category	Category	Enrolled in health insurance (%)	Women having Insurance (%)	Women not having Insurance (%)
Visited Health Facility in Last Year	Visited	11.77	75.77	68.80
	Didn’t Visit	8.60	24.23	31.20
Time to Nearest Health Facility	15 Minutes or Less	11.90	75.75	67.95
	16 to 30 Minutes	9.51	17.83	20.55
	31 to 60 Minutes	6.71	4.37	7.37
	Over 1 Hour	5.67	2.05	4.13
How Travel to Nearest Health Facility	Motorized	16.76	17.32	10.42
	Not Motorized	10.06	82.68	89.58
Type of Nearest Health Facility	Health Post	8.72	32.10	40.72
	Basic Health Care Center	14.80	6.20	4.33
	Community Health Unit	7.97	1.85	2.59
	Government Hospital	18.56	7.45	3.96
	Other	12.92	0.72	0.59
	Pharmacy	9.90	19.09	21.05
	Primary Health Center	3.96	0.53	1.55
	Private Clinic	12.98	25.24	20.51
	Private Hospital	13.51	4.26	3.31
	Urban Health Center	18.23	2.55	1.38

Source: Nepal Demographic and Health Survey (NDHS) 2022

Most of the women live within 15 minutes of a health facility. The share of women with insurance is highest for those closest to a facility. These declines as the time to the facility increases. Those who take a motorized form of transport to a health facility have higher rates of insurance (17%) compared to the non-motorized transport (10%), although most of the women use a non-motorized form of transport to reach it, regardless of their insurance status. Women who live near a government hospital or urban health center have the highest rates of insurance coverage, and the lowest insurance coverage is among women whose nearest facility is a primary health center.

Table 4 presents the data on the relationship between women’s health insurance status and the extent to which they face difficulty obtaining health care. They were asked if each of the four variables–permission to go for treatment, obtaining money for treatment, distance to a health facility, and not wanting to go alone—was a “big problem” or “not big problem.” As the results showed, 10% of the women find obtaining permission for treatment to be a big problem, while 17% having no insurance perceive it as a big problem. 23% of the women having insurance report obtaining money for treatment as a big problem, both high numbers, although over 50% higher for women without insurance. Distance to the facility is a greater obstacle for the women without insurance (39%) than the women with insurance (25%), which reflects that, women with insurance live closer to facilities than those without insurance. Both groups do not want to go alone to a facility, so they find going alone as a big problem. A simple majority (56%) of the women without insurance reported it as a big problem.

**Table 4:** Health insurance enrollment and problem care.

Variables	Category	Women having Insurance (%)	Women having no Insurance (%)
Getting Permission to go for Treatment	Big Problem	10.04	16.85
	Not Big Problem	89.96	83.15
Getting Money for Treatment	Big Problem	23.05	36.79
	Not Big Problem	76.95	63.21
Distance to Health Facility	Big Problem	25.48	38.60
	Not Big Problem	74.52	61.40
Not Wanting to Go Alone	Big Problem	44.03	56.18
	Not Big Problem	55.97	43.82

Source: Nepal Demographic and Health Survey (NDHS) 2022

### 3.2 Association of socio-demographic variables and enrollment in government health insurance program

Table 5 shows that the men who resided outside of Koshi Province have lower rates of the government-funded health insurance compared to those who lived in Koshi Province. This finding remains after controlling all other socio-demographic characteristics. Concerning residence, while rural men are less likely to have insurance than urban men, this relationship was not maintained when adding the control variables. In the bivariate regression, all castes are less likely to have insurance than the Hill Brahmin/Chhetri. With the multivariate relationship, there is no statistical difference between the Dalit and the Hill Brahmin/Chhetri, although the other castes remain lower but statistically significant. The ’Never in union’ men have lower rates of insurance than the married men, but this relationship is not statistically different when controlling the other variables. The education differences seen in the descriptive statistics hold true when testing for significance, both with and without control variables. Looking at wealth, compared to the middle quintile, the poorest residents are less likely to have insurance, and the richest group are more likely to have insurance, although the second and fourth quintiles are not statistically significant differences from the middle. There is no significant difference across the household size. The only difference from the reference age group (age 30–34) is age 35–39, as appeared statistically significant. There is no statistically significant difference in insurance coverage for the self-rated health categories, compared to those who report being in good health. Finally, although there is a difference in insurance with employment status, when controlling for the other variables, only the manual labor category has lower insurance coverage than those who are employed (in non-agricultural and non-manual labor).

**Table 5:** Regression analysis among socio-demographic characters and having government health insurance (Men).

Category	Variable	Bivariate OR (95%CI)	Multivariate AOR (95%CI)
Province	Bagmati	0.33 *** (0.20–0.53)	0.18 ***(0.11–0.31)
	Gandaki	0.47 *** (0.29–0.77)	0.29 *** (0.17–0.49)
	Karnali	0.50 *** (0.30–0.84)	0.37 *** (0.20–0.68)
	Koshi	1 (ref)	1 (ref)
	Lumbini	0.36 *** (0.20–0.62)	0.23 *** (0.13–0.40)
	Madhesh	0.12 ***(0.06–0.21)	0.12 ***(0.06–0.23)
	Sudurpashchim	0.28 ***(0.14–0.56)	0.23 ***(0.11–0.51)
Residence	Rural	0.74 * (0.54–1.02)	1.08 (0.78–1.50)
	Urban	1 (ref)	1 (ref)
Caste	Dalit	0.53 ***(0.36–0.78)	1.32(0.88–1.97)
	Hill Brahmin Chhetri	1 (ref)	1 (ref
	Janajatis	0.47 ***(0.33–0.66)	0.57 ***(0.40–0.82)
	Muslim/Others	0.30 ***(0.12–0.70)	0.51 *(0.24–1.07)
	Terai/Madhesi	0.25 ***(0.15–0.41)	0.47 ***(0.27–0.81)
Marital Status	Formerly in Union	0.73(0.24–2.20)	0.77(0.22–2.61)
	In Union	1 (ref)	1 (ref)
	Never in Union	0.80 **(0.66–0.98)	0.78(0.57–1.07)
Education	Basic	1 (ref)	1 (ref)
	Higher	3.87 ***(2.66–5.65)	2.74 ***(1.77–4.23)
	None	0.38 ***(0.19–0.78)	0.46*(0.21–1.03)
	Secondary	2.19 ***(1.69–2.84)	1.87 ***(1.40–2.50)
Wealth	Middle	1 (ref)	1 (ref)
	Poorer	1.00(0.70–1.42)	0.89(0.59–1.33)
	Poorest	0.60 **(0.39–0.92)	0.42 ***(0.26–0.69)
	Richer	1.35(0.92–1.98)	1.1(0.74–1.65)
	Richest	2.20 ***(1.49–3.26)	1.61 **(1.08–2.38)
De Jure Household Size	1 Member	0.97(0.45–2.1)	1.06(0.42–2.65)
	2 Members	1.08(0.68–1.72)	1.12(0.69–1.83)
	3 Members	1.10(0.77–1.56)	1.06(0.73–1.54)
	4 Members	1 (ref)	1 (ref)
	5 Members	1.09(0.73–1.64)	1.18(0.78–1.77)
	6 Members	1.24(0.82–1.88)	1.28(0.82–2.01)
	7+ Members	0.91(0.58–1.42)	1.48(0.88–2.48)
Age	15–19	0.64 **(0.44–0.94)	0.73(0.45–1.19)
	20–24	1.13(0.75–1.71)	1.28(0.83–1.99)
	25–29	0.88(0.58–1.32)	0.94(0.61–1.43)
	30–34	1 (ref)	1 (ref)
	35–39	1.50 **(1.02–2.21)	1.80 ***(1.2–2.71)
	40–44	1.12(0.74–1.7)	1.25(0.81–1.95)
	45–49	1.27(0.84–1.94)	1.32(0.85–2.06)
Self-rated Health	Bad/Very Bad	1.16(0.72–1.87)	1.37(0.82–2.31)
	Good	1 (ref)	1 (ref)
	Moderate	0.92(0.72–1.18)	1.10(0.88–1.39)
	Very Good	0.67(0.41–1.10)	0.75(0.45–1.24)
Occupation	Agriculture	0.60 ***(0.45–0.82)	0.87(0.61–1.23)
	Employed	1 (ref)	1 (ref)
	Manual	0.39 *** (0.29–0.53)	0.69 **(0.50–0.95)
	Unemployed		
	Others	0.68 **(0.47–0.99)	1.47(0.91–2.37)

Note: *** = Significant at p<0.001;

** = p<0.01, and

* = p<0.05

Table 6 also shows that, like the men, the women who resided in other provinces are less likely to have health insurance compared to the women who resided in the Koshi Province. As regards caste, all groups have lower insurance coverage compared to the Hill Brahmin/Chhetri, both with and without the control variables. As with the men, the residence was significant in the bivariate analysis, with the rural women being less likely to have government insurance than urban women, although this relationship does not sustain when adding control variables. There is no significant difference in the insurance status by marital status, although there is a strong positive relationship between education and insurance coverage. Compared to women in the middle wealth quintile households, the poorest women were less likely to have insurance, while the richer and richest quintiles were most likely to have insurance. As with the men, there is no relationship found between household size and insurance coverage when controlling the other variables. Age patterns and insurance are relevant for the women, unlikely the men; the younger women have lower odds of having insurance compared to women aged 30–34, and the older women are more likely to have insurance. With self-rated health, women with bad or very bad health were more likely to have insurance than the women with good health.

**Table 6:** Association of socio-demographic characters and having health insurance (Women).

Variables	Category	Bivariate OR (95% CI)	Multivariate AOR (95% CI)
Province	Koshi	1 (ref)	1 (ref)
	Madhesh	0.11 *** (0.06–0.19)	0.15 ***(0.08–0.27)
	Bagmati	0.50 *** (0.33–0.77)	0.29 *** (0.19–0.44)
	Gandaki	0.78 (0.53–1.13)	0.57 ***(0.39–0.83)
	Lumbini	0.40 ***(0.26–0.63)	0.35 ***(0.23–0.53)
	Karnali	0.45 ***(0.29–0.7)	0.51 ***(0.32–0.81)
	Sudurpashchim	0.28 ***(0.15–0.5)	0.26 ***(0.13–0.52)
Residence	Urban	1 (ref)	1 (ref)
	Rural	0.61 ***(0.47–0.79)	1.01(0.77–1.31)
Caste	Hill Brahmin Chhetri	1 (ref)	1 (ref)
	Terai/Madhesi	0.26 *** (0.18–0.37)	0.53 ***(0.35–0.79)
	Dalit	0.37 ***(0.28–0.49)	0.76 *(0.57–1.02)
	Janajatis	0.49 ***(0.38–0.63)	0.54 ***(0.42–0.7)
	Muslim/Others	0.16 ***(0.09–0.31)	0.40 ***(0.21–0.76)
Marital Status	In Union	1 (ref)	1 (ref)
	Never In Union	1.01(0.87–1.18)	1.03(0.8–1.31)
	Formerly In Union	0.95(0.68–1.32)	0.93(0.65–1.35)
Education	None	0.58 ***(0.45–0.75)	0.66 ***(0.5–0.87)
	Basic	1 (ref)	1 (ref)
	Secondary	2.36 ***(1.98–2.83)	2.02 ***(1.67–2.44)
	Higher	4.16 ***(3.13–5.53)	2.42 ***(1.84–3.18)
Wealth	Poorest	0.50 ***(0.37–0.67)	0.43 ***(0.31–0.6)
	Poorer	0.92(0.71–1.19)	0.92(0.7–1.21)
	Middle	1 (ref)	1 (ref)
	Richer	1.73 ***(1.36–2.19)	1.52 ***(1.19–1.95)
	Richest	2.87 ***(2.09–3.94)	1.93 ***(1.45–2.56)
De Jure Household Size	1 Member	0.81(0.5–1.32)	0.86(0.52–1.43)
	2 Members	0.92(0.7–1.21)	0.88(0.67–1.15)
	3 Members	1.02(0.82–1.27)	0.99(0.79–1.25)
	4 Members	1 (ref)	1 (ref)
	5 Members	1.14(0.9–1.44)	1.34 **(1.06–1.70)
	6 Members	0.92(0.71–1.18)	1.15(0.89–1.50)
	7+ Members	0.74 **(0.56–0.99)	1.21(0.9–1.62)
Age	15–19	0.64 ***(0.49–0.84)	0.72 *(0.51–1.02)
	20–24	0.78 **(0.62–0.98)	0.75 **(0.58–0.96)
	25–29	0.99(0.8–1.23)	0.95(0.75–1.19)
	30–34	1 (ref)	1 (ref)
	35–39	0.92(0.73–1.15)	1.08(0.85–1.37)
	40–44	1.06(0.81–1.38)	1.48 ***(1.12–1.96)
	45–49	1.07(0.84–1.35)	1.57 ***(1.18–2.09)
Self-rated Health	Very Good	0.64 **(0.42–0.98)	1.07(0.70–1.64)
	Good (ref)	1 (ref)	1 (ref)
	Moderate	1.40 ***(1.17–1.66)	1.10(0.92–1.31)
	Bad/Very Bad	1.50 ***(1.19–1.89)	1.68 ***(1.34–2.11)
Occupation	Employed	1 (ref)	1 (ref)
	Agriculture	0.33 ***(0.27–0.42)	0.69 ***(0.56–0.86)
	Manual	0.35 ***(0.25–0.49)	0.68 **(0.49–0.94)
	Unemployed/Others	0.37 ***(0.29–0.48)	0.54 ***(0.42–0.68)
Migration	Husband Staying Elsewhere	0.71 ***(0.60–0.83)	0.88(0.74–1.04)
	Husband Living with Her	1 (ref)	1 (ref)

Note: *** = Significant at p<0.001;

** = p<0.01, and

* = p<0.05

As regards the married women, those women whose husbands are staying elsewhere are less likely to have insurance than women whose husbands live with them, as shown by the bivariate analysis, although there is no statistical difference when controlling for the other variables.

**Fig 1:**
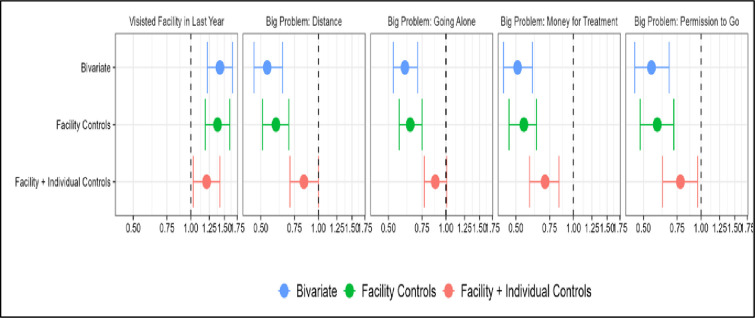
Association of likelihood of visiting a health facility in the last year and having big problems accessing care, based on coverage insurance status.

Unlike previous analyses that examined insurance coverage alone, Fig 1 explores the relationship between possessing health insurance and accessing healthcare services. The bivariate analysis reveals that the women with government insurance are 42% more likely to visit a health facility in the last year compared to those women who do not. After adding controls for the distance to a facility and the means of transport to the facility, having insurance is still positively and statistically associated with visiting the health facility. With the addition of sociodemographic control variables, having insurance is associated with a 21% increase in the odds of visiting the health facility compared to those who do not have insurance. It is important to emphasize that insurance is a current status measure, while visiting a health facility is a retrospective measure (Fig 1).

For all questions about problems accessing care (permission to go for treatment, obtaining money for treatment, distance to the health facility, and not wanting to go alone), the women with insurance are less likely to report major problems than women without insurance in all four cases, even after controlling for distance, mode of transport to the facility, and socioeconomic demographics [S1 Table].

## 4 Discussions

We found that almost one out of ten (12.0% women and 13.3% men) of the total respondents had any health insurance. Of them, 10.8% of the women and 10.2% of the men were enrolled in the government health insurance scheme [17], which is much lower than the 60% coverage goal for 2023. The survey also finds a lower insurance coverage than the Health Insurance Board (HIB) claims (32% of total households and 5.9 million people, which makes roughly 20% of the population enrolled in the government HIP within the 6th year of its operation) [3]. This does not mean that all participants have continued to re-enroll annually. The NDHS 2022 does not allow us to assess dropout and re-enrollment rates. The data from the NDHS 2022 reveal that having health insurance varied according to age, gender, caste, residence setting, geographic areas, occupation, and wealth status, although no subgroup was near 30%, which indicates that socio-demographic disparities exist in health insurance [23]. Issues with inequalities exist, although the largest problem is the overall low coverage of the government (or any other type of) health insurance. As we have shown, insurance is associated with an increased likelihood of visits and fewer problems accessing care. Thus, for Nepal, the expansion of health insurance is a pressing need.

The health insurance program was launched without adequate preparation. It requires comprehensive information, counseling, and education. Despite this, many people still lack knowledge about enrollment, service access, program limitations, service availability, and referral procedures [13]. While people often perceive health insurance as a panacea, the program has constraints such as coverage limits, specific services included, and available healthcare resources. Effective communication about the program is crucial for its success [24, 25].

In this research, several issues associated with health insurance enrollment were identified. Most of the sociodemographic characteristics of the respondents were associated with having health insurance, which is similar to previous studies [16, 26, 27]. One of the most striking findings is the regional variable, with the respondents from Koshi Province (20%–22%) having more health insurance compared to those from the Madhesh Province (2%–3%). Similarly, Dalit and Muslim women had lower health insurance coverage compared to the others. Although the policy exists to ensure that no one should be left behind for healthcare services based on their economic status, the poorest residents are less likely to have health insurance in Nepal. A major intervention is needed to increase people’s participation in the government HIP to achieve universal health coverage.

We found that several sociodemographic factors were significantly associated with health insurance coverage. Similar findings were observed in other studies from different countries, including Nepal [27–33]. Most of the studies showed that the richer residents are more likely to have health insurance [28–30, 33, 34] and the same was found in this study, too. However, the poor residents are also more likely to have health insurance in Ghana [35]. The self-rated health status as ‘good’ is considered a barrier to having insurance [35, 36]. In addition, as facilitators for having health insurance [36, 37], the pro-public health policies were also examined. Another similar results have shown in India that 51% of males and 49% of females were enrolled in health insurance, but female-headed households appeared more likely to enroll in health insurance [38].

Health insurance has multiple benefits beyond health and well-being. It can improve the overall economy, healthcare efficiency, and quality of care [39–41]. While there are challenges to implementing the health insurance, it is crucial to address these and move forward with the program as progress is currently lagging behind the targets of universal health coverage. Health insurance is seen as a tool for broader societal improvement, not just better health outcomes [42, 43]. This study found the associated factors for enrollment in health insurance where the intervention can be focused for betterment.

The NDHS 2022 reveals a disappointing finding that a large proportion of active, informed, and civically engaged residents aged 15 to 49 are not enrolled in the government HIP. Several interventions could lead to higher enrollment, such as awareness campaigns [44], quality services for the enrolled members, reminders to households to renew their insurance, proper management of patients’ complaints, user-friendly services, higher coverage (ceiling amount), and pro-poor policies [19, 26, 27, 45]. Enacting pro-participant rules and regulations can also increase satisfaction with the program, which can lead to higher participation.

### 4.1 Strengths and limitations

Since the NDHS 2022 was a nationally representative sample survey, the findings can be applied to the nation as a whole. While government health insurance is purchased for the household, our analysis was restricted to individuals age 15–49 because the health insurance status was asked only in the men’s and women’s questionnaires. Some individuals may not be aware that their household has government insurance, which would possibly underestimate the reports of insurance coverage. Additionally, since we used the secondary data, some data were missed, particularly about whether they utilized services under the insurance scheme or not, what kinds of services they utilized, and if there was a large dropout of the insurance program. These results can be used for policy interventions while recognizing the limitations discussed above.

## 5 Conclusions

Nepal’s National Health Insurance Scheme has not reached the level of implementation expected in 2022. It shows that there are provincial and geographical variances, including the poor-rich variance. The government of Nepal must work to identify the barriers to participation of the households in order to increase the use of health insurance. School-based and community-based awareness campaigns might be a better option to sensitize the people for better understanding, participation, and renewal. Areas characterized by elevated levels of non-enrollment rate should be prioritized for focused intervention. Moreover, policymakers should take these findings into account during intervention planning and implementation.

## Supporting information

S1 Table Association of socio-demographic characteristics and enrollment in health insurance. (DOCX)
