# Supplementary material for: Enrollment and associated factors of the national health insurance program of Nepal: Further analysis of the Nepal Demographic and Health Survey 2022
Source: PLoS One. 2024 Oct 3;19(10):e0310324. doi: 10.1371/journal.pone.0310324 (PMC11449327; doi:10.1371/journal.pone.0310324)
Supplement: S1 Table — (DOCX) [file pone.0310324.s001.docx]

APPENDICES

S1 Appendix Association of likelihood of visiting a health facility in the last year

|  | Category | Visited health facility in last year | | |
| --- | --- | --- | --- | --- |
| Variables |  | Bivariate OR  [95% CI] | Multivariate AOR 1  [95% CI] | Multivariate AOR 2  [95% CI] |
| Insurance status | Has government insurance | 1.42 *** [1.22-1.65] | 1.38 *** [1.19-1.6] | 1.21 ** [1.03-1.42] |
|  | No government insurance [ref] | 1 [ref] | 1 [ref] | 1 [ref] |
| Travel time to health facility | 0 to 15 minutes [ref] |  | 1 [ref] | 1 [ref] |
|  | 16 to 30 minutes |  | 0.87 ** [0.78-0.98] | 0.85 ** [0.75-0.97] |
|  | 31 to 60 minutes |  | 0.96 [0.82-1.12] | 0.93 [0.78-1.1] |
|  | Over 1 hour |  | 0.75 *** [0.6-0.93] | 0.69 *** [0.54-0.88] |
| Transport to health facility | Motorized travel to facility [ref] |  | 1 [ref] | 1 [ref] |
|  | Non-motorized travel to facility |  | 0.77 *** [0.67-0.89] | 0.87 * [0.75-1] |
| Province | Koshi [ref] |  |  | 1 [ref] |
|  | Madhesh |  |  | 0.9 [0.7-1.15] |
|  | Bagmati |  |  | 0.93 [0.77-1.14] |
|  | Gandaki |  |  | 0.95 [0.77-1.17] |
|  | Lumbini |  |  | 1.09 [0.91-1.31] |
|  | Karnali |  |  | 0.84 [0.68-1.04] |
|  | Sudurpashchim |  |  | 1.14 [0.91-1.43] |
| Residence | Urban [ref] |  |  | 1 [ref] |
|  | Rural |  |  | 1 [0.88-1.13] |
| Caste | Hill Brahmin/Chhetri [ref] |  |  | 1 [ref] |
|  | Terai/Madheshi |  |  | 0.77 ** [0.61-0.96] |
|  | Dalit |  |  | 1 [0.85-1.17] |
|  | Janajatis |  |  | 0.85 ** [0.74-0.97] |
|  | Muslim/others |  |  | 0.92 [0.68-1.25] |
| Marital status | In union [ref] |  |  | 1 [ref] |
|  | Never In union |  |  | 0.26 *** [0.22-0.31] |
|  | Formerly In union |  |  | 0.77 ** [0.61-0.98] |
| Education | None |  |  | 0.76 *** [0.68-0.87] |
|  | Basic [ref] |  |  | 1 [ref] |
|  | Secondary |  |  | 1.25 *** [1.1-1.41] |
|  | Higher |  |  | 1.18 [0.9-1.55] |
| Wealth | Poorest |  |  | 0.93 [0.79-1.1] |
|  | Poorer |  |  | 1 [0.88-1.14] |
|  | Middle [ref] |  |  | 1 [ref] |
|  | Richer |  |  | 1.04 [0.9-1.2] |
|  | Richest |  |  | 1.02 [0.85-1.22] |
| De jure household size | 1 member |  |  | 0.86 [0.63-1.16] |
|  | 2 members |  |  | 0.92 [0.78-1.09] |
|  | 3 members |  |  | 0.97 [0.85-1.1] |
|  | 4 members [ref] |  |  | 1 [ref] |
|  | 5 members |  |  | 0.93 [0.81-1.05] |
|  | 6 members |  |  | 0.99 [0.85-1.17] |
|  | 7+ members |  |  | 0.94 [0.82-1.09] |
| Age | 15–19 |  |  | 1.04 [0.86-1.26] |
|  | 20–24 |  |  | 1.53 *** [1.29-1.82] |
|  | 25–29 |  |  | 1.46 *** [1.22-1.73] |
|  | 30–34 [ref] |  |  | 1 [ref] |
|  | 35–39 |  |  | 0.84 ** [0.71-0.99] |
|  | 40–44 |  |  | 0.59 *** [0.49-0.7] |
|  | 45–49 |  |  | 0.6 *** [0.49-0.73] |
| Self rates health | Very good |  |  | 1.08 [0.86-1.34] |
|  | Good [ref] |  |  | 1 [ref] |
|  | Moderate |  |  | 1.13 ** [1.02-1.26] |
|  | Bad/very bad |  |  | 1.76 *** [1.47-2.09] |
| Occupation | Employed [ref] |  |  | 1 [ref] |
|  | Agriculture |  |  | 0.98 [0.84-1.14] |
|  | Manual |  |  | 0.97 [0.78-1.2] |
|  | Unemployed/others |  |  | 0.95 [0.82-1.1] |
|  | | | | |
| Note: *** = Significant at p<0.001; **=p<0.01, and *=p<0.05 | | | | |
|  | | | | |

S2 Appendix Association of having a major problem obtaining permission to go for treatment

|  | Category | Obtaining permission to go for treatment is a big problem | | |
| --- | --- | --- | --- | --- |
| Variables |  | Bivariate OR  [95% CI] | Multivariate AOR 1  [95% CI] | Multivariate AOR 2  [95% CI] |
| Insurance status | Has government insurance | 0.55 *** [0.45-0.68] | 0.59 *** [0.48-0.72] | 0.78 ** [0.63-0.96] |
|  | No government insurance [ref] | 1 [ref] | 1 [ref] | 1 [ref] |
| Travel time to health facility | 0 to 15 minutes [ref] |  | 1 [ref] | 1 [ref] |
|  | 16 to 30 minutes |  | 2.26 *** [1.94-2.65] | 1.53 *** [1.3-1.79] |
|  | 31 to 60 minutes |  | 2.64 *** [2.13-3.27] | 1.57 *** [1.26-1.95] |
|  | Over 1 hour |  | 2.75 *** [2.06-3.67] | 1.29 * [0.97-1.7] |
| Transport to health facility | Motorized travel to facility [ref] |  | 1 [ref] | 1 [ref] |
|  | Non-motorized travel to facility |  | 0.96 [0.75-1.23] | 0.84 [0.66-1.08] |
| Province | Koshi [ref] |  |  | 1 [ref] |
|  | Madhesh |  |  | 0.48 *** [0.34-0.68] |
|  | Bagmati |  |  | 0.82 [0.6-1.12] |
|  | Gandaki |  |  | 0.58 *** [0.4-0.85] |
|  | Lumbini |  |  | 0.63 *** [0.45-0.88] |
|  | Karnali |  |  | 1.76 *** [1.33-2.33] |
|  | Sudurpashchim |  |  | 0.38 *** [0.26-0.55] |
| Residence | Urban [ref] |  |  | 1 [ref] |
|  | Rural |  |  | 1.17 [0.96-1.42] |
| Caste | Hill Brahmin/Chhetri [ref] |  |  | 1 [ref] |
|  | Terai/Madheshi |  |  | 1.13 [0.84-1.51] |
|  | Dalit |  |  | 1.14 [0.93-1.41] |
|  | Janajatis |  |  | 1.1 [0.93-1.31] |
|  | Muslim/others |  |  | 1.07 [0.73-1.59] |
| Marital status | In union [ref] |  |  | 1 [ref] |
|  | Never In union |  |  | 0.58 *** [0.48-0.69] |
|  | Formerly In union |  |  | 0.61 *** [0.44-0.84] |
| Education | None |  |  | 1.1 [0.94-1.28] |
|  | Basic [ref] |  |  | 1 [ref] |
|  | Secondary |  |  | 0.51 *** [0.44-0.59] |
|  | Higher |  |  | 0.42 *** [0.24-0.72] |
| Wealth | Poorest |  |  | 1.45 *** [1.18-1.78] |
|  | Poorer |  |  | 1.3 *** [1.1-1.54] |
|  | Middle [ref] |  |  | 1 [ref] |
|  | Richer |  |  | 0.86 [0.71-1.05] |
|  | Richest |  |  | 0.57 *** [0.44-0.75] |
| De jure household size | 1 member |  |  | 1.09 [0.67-1.77] |
|  | 2 members |  |  | 0.83 * [0.68-1.02] |
|  | 3 members |  |  | 0.99 [0.84-1.16] |
|  | 4 members [ref] |  |  | 1 [ref] |
|  | 5 members |  |  | 1.06 [0.9-1.25] |
|  | 6 members |  |  | 1.03 [0.86-1.23] |
|  | 7+ members |  |  | 1.31 *** [1.11-1.54] |
| Age | 15–19 |  |  | 1.64 *** [1.31-2.06] |
|  | 20–24 |  |  | 1.53 *** [1.26-1.85] |
|  | 25–29 |  |  | 1.19 * [0.97-1.45] |
|  | 30–34 [ref] |  |  | 1 [ref] |
|  | 35–39 |  |  | 1.02 [0.84-1.25] |
|  | 40–44 |  |  | 1.13 [0.92-1.39] |
|  | 45–49 |  |  | 1.32 ** [1.07-1.65] |
| Self rates health | Very good |  |  | 1.16 [0.88-1.54] |
|  | Good [ref] |  |  | 1 [ref] |
|  | Moderate |  |  | 1.01 [0.88-1.16] |
|  | Bad/very bad |  |  | 1.2 ** [1-1.45] |
| Occupation | Employed [ref] |  |  | 1 [ref] |
|  | Agriculture |  |  | 1.46 *** [1.17-1.81] |
|  | Manual |  |  | 1.72 *** [1.3-2.28] |
|  | Unemployed/others |  |  | 1.62 *** [1.26-2.08] |
|  | | | | |
| Note: *** = Significant at p<0.001; **=p<0.01, and *=p<0.05 | | | | |

S3 Appendix Association of having a major problem obtaining money to go for treatment

|  |  | Money for treatment is a big problem | | |
| --- | --- | --- | --- | --- |
| Variables | Category | Bivariate OR  [95% CI] | Multivariate AOR 1  [95% CI] | Multivariate AOR 2  [95% CI] |
| Insurance status | Has government insurance | 0.51 *** [0.43-0.61] | 0.55 *** [0.46-0.64] | 0.71 *** [0.59-0.84] |
|  | No government insurance [ref] | 1 [ref] | 1 [ref] | 1 [ref] |
| Travel time to health facility | 0 to 15 minutes [ref] |  | 1 [ref] | 1 [ref] |
|  | 16 to 30 minutes |  | 1.96 *** [1.72-2.24] | 1.37 *** [1.2-1.55] |
|  | 31 to 60 minutes |  | 2.09 *** [1.74-2.5] | 1.33 *** [1.1-1.61] |
|  | Over 1 hour |  | 2.12 *** [1.6-2.81] | 1.09 [0.77-1.53] |
| Transport to health facility | Motorized travel to facility [ref] |  | 1 [ref] | 1 [ref] |
|  | Non-motorized travel to facility |  | 1.25 ** [1.02-1.54] | 1.11 [0.89-1.37] |
| Province | Koshi [ref] |  |  | 1 [ref] |
|  | Madhesh |  |  | 0.71 ** [0.54-0.95] |
|  | Bagmati |  |  | 0.68 ** [0.5-0.92] |
|  | Gandaki |  |  | 0.42 *** [0.29-0.62] |
|  | Lumbini |  |  | 0.58 *** [0.42-0.79] |
|  | Karnali |  |  | 1.23 [0.88-1.72] |
|  | Sudurpashchim |  |  | 0.58 *** [0.43-0.78] |
| Residence | Urban [ref] |  |  | 1 [ref] |
|  | Rural |  |  | 0.99 [0.83-1.18] |
| Caste | Hill Brahmin/Chhetri [ref] |  |  | 1 [ref] |
|  | Terai/Madheshi |  |  | 1.25 ** [1-1.56] |
|  | Dalit |  |  | 1.38 *** [1.16-1.64] |
|  | Janajatis |  |  | 1.16 * [1-1.34] |
|  | Muslim/others |  |  | 1.19 [0.87-1.65] |
| Marital status | In union [ref] |  |  | 1 [ref] |
|  | Never In union |  |  | 0.9 [0.76-1.06] |
|  | Formerly In union |  |  | 1.51 *** [1.2-1.91] |
| Education | None |  |  | 1.15 ** [1.02-1.3] |
|  | Basic [ref] |  |  | 1 [ref] |
|  | Secondary |  |  | 0.6 *** [0.53-0.68] |
|  | Higher |  |  | 0.31 *** [0.22-0.43] |
| Wealth | Poorest |  |  | 1.4 *** [1.15-1.69] |
|  | Poorer |  |  | 1.25 *** [1.1-1.42] |
|  | Middle [ref] |  |  | 1 [ref] |
|  | Richer |  |  | 0.85 ** [0.74-0.99] |
|  | Richest |  |  | 0.54 *** [0.43-0.67] |
| De jure household size | 1 member |  |  | 0.86 [0.61-1.2] |
|  | 2 members |  |  | 0.94 [0.8-1.09] |
|  | 3 members |  |  | 1.05 [0.93-1.2] |
|  | 4 members [ref] |  |  | 1 [ref] |
|  | 5 members |  |  | 1.08 [0.95-1.24] |
|  | 6 members |  |  | 0.96 [0.84-1.11] |
|  | 7+ members |  |  | 1.05 [0.92-1.21] |
| Age | 15–19 |  |  | 0.89 [0.74-1.07] |
|  | 20–24 |  |  | 0.99 [0.85-1.15] |
|  | 25–29 |  |  | 1 [0.85-1.17] |
|  | 30–34 [ref] |  |  | 1 [ref] |
|  | 35–39 |  |  | 1.04 [0.89-1.22] |
|  | 40–44 |  |  | 1.18 * [1-1.4] |
|  | 45–49 |  |  | 1.29 ** [1.06-1.56] |
| Self rates health | Very good |  |  | 0.51 *** [0.39-0.67] |
|  | Good [ref] |  |  | 1 [ref] |
|  | Moderate |  |  | 1.17 *** [1.04-1.31] |
|  | Bad/very bad |  |  | 1.49 *** [1.26-1.76] |
| Occupation | Employed [ref] |  |  | 1 [ref] |
|  | Agriculture |  |  | 1.23 ** [1.04-1.46] |
|  | Manual |  |  | 1.49 *** [1.2-1.84] |
|  | Unemployed/others |  |  | 1.27 *** [1.07-1.49] |
|  | | | | |
| Note: ** = Significant at p<0.001; **=p<0.01, and *=p<0.05 | | | | |
|  | | | | |
|  | | | | |

S4 Appendix Association of distance to a health facility being a major problem for treatment

|  | Category | Distance to health facility is a big problem | | |
| --- | --- | --- | --- | --- |
| Variables |  | Bivariate OR  [95% CI] | Multivariate AOR 1 [95% CI] | Multivariate AOR 2  [95% CI] |
| Insurance status | Has government insurance | 0.54 *** [0.46-0.65] | 0.6 *** [0.51-0.7] | 0.84 ** [0.71-1] |
|  | No government insurance [ref] | 1 [ref] | 1 [ref] | 1 [ref] |
| Travel time to health facility | 0 to 15 minutes [ref] |  | 1 [ref] | 1 [ref] |
|  | 16 to 30 minutes |  | 3.45 *** [2.97-3.99] | 2.48 *** [2.14-2.88] |
|  | 31 to 60 minutes |  | 9.48 *** [7.8-11.53] | 6.72 *** [5.48-8.24] |
|  | Over 1 hour |  | 25.86 *** [18.54-36.07] | 15.02 *** [10.42-21.64] |
| Transport to health facility | Motorized travel to facility [ref] |  | 1 [ref] | 1 [ref] |
|  | Non-motorized travel to facility |  | 1.06 [0.88-1.27] | 0.88 [0.73-1.06] |
| Province | Koshi [ref] |  |  | 1 [ref] |
|  | Madhesh |  |  | 1.26 [0.94-1.69] |
|  | Bagmati |  |  | 0.65 *** [0.49-0.85] |
|  | Gandaki |  |  | 0.61 *** [0.44-0.85] |
|  | Lumbini |  |  | 0.54 *** [0.4-0.72] |
|  | Karnali |  |  | 1.14 [0.85-1.53] |
|  | Sudurpashchim |  |  | 0.35 *** [0.25-0.49] |
| Residence | Urban [ref] |  |  | 1 [ref] |
|  | Rural |  |  | 1.17 * [0.99-1.39] |
| Caste | Hill Brahmin/Chhetri [ref] |  |  | 1 [ref] |
|  | Terai/Madheshi |  |  | 1.21 [0.95-1.54] |
|  | Dalit |  |  | 1.14 [0.94-1.37] |
|  | Janajatis |  |  | 1.06 [0.91-1.24] |
|  | Muslim/others |  |  | 1.35 [0.94-1.96] |
| Marital status | In union [ref] |  |  | 1 [ref] |
|  | Never In union |  |  | 0.95 [0.82-1.1] |
|  | Formerly In union |  |  | 1 [0.78-1.28] |
| Education | None |  |  | 1.12 * [0.98-1.27] |
|  | Basic [ref] |  |  | 1 [ref] |
|  | Secondary |  |  | 0.7 *** [0.61-0.8] |
|  | Higher |  |  | 0.47 *** [0.34-0.67] |
| Wealth | Poorest |  |  | 1.84 *** [1.52-2.23] |
|  | Poorer |  |  | 1.39 *** [1.21-1.58] |
|  | Middle [ref] |  |  | 1 [ref] |
|  | Richer |  |  | 0.81 *** [0.69-0.95] |
|  | Richest |  |  | 0.52 *** [0.42-0.65] |
| De jure household size | 1 member |  |  | 0.88 [0.65-1.2] |
|  | 2 members |  |  | 0.91 [0.78-1.07] |
|  | 3 members |  |  | 1.01 [0.89-1.15] |
|  | 4 members [ref] |  |  | 1 [ref] |
|  | 5 members |  |  | 0.94 [0.83-1.08] |
|  | 6 members |  |  | 0.96 [0.83-1.11] |
|  | 7+ members |  |  | 0.96 [0.84-1.11] |
| Age | 15–19 |  |  | 0.89 [0.74-1.07] |
|  | 20–24 |  |  | 0.99 [0.85-1.14] |
|  | 25–29 |  |  | 0.94 [0.79-1.13] |
|  | 30–34 [ref] |  |  | 1 [ref] |
|  | 35–39 |  |  | 1.02 [0.86-1.2] |
|  | 40–44 |  |  | 1.11 [0.93-1.32] |
|  | 45–49 |  |  | 1.21 ** [1-1.47] |
| Self rates health | Very good |  |  | 0.52 *** [0.37-0.72] |
|  | Good [ref] |  |  | 1 [ref] |
|  | Moderate |  |  | 1.23 *** [1.08-1.39] |
|  | Bad/very bad |  |  | 1.74 *** [1.44-2.1] |
| Occupation | Employed [ref] |  |  | 1 [ref] |
|  | Agriculture |  |  | 1.32 *** [1.11-1.57] |
|  | Manual |  |  | 1.15 [0.91-1.46] |
|  | Unemployed/others |  |  | 1.17 [0.97-1.4] |
|  | | | | |
| Note: *** = Significant at p<0.001; **=p<0.01, and *=p<0.05 | | | | |
|  | | | | |
|  | | | | |

S5 Appendix Association of not wanting to go alone being a big problem for treatment

|  | Category | Not wanting to go alone is a big problem | | |
| --- | --- | --- | --- | --- |
| Variables |  | Bivariate OR  [95% CI] | Multivariate AOR 1  [95% CI] | Multivariate AOR 2  [95% CI] |
| Insurance status | Has government insurance | 0.61 *** [0.53-0.71] | 0.65 *** [0.57-0.75] | 0.88 * [0.77-1.01] |
|  | No government insurance [ref] | 1 [ref] | 1 [ref] | 1 [ref] |
| Travel time health facility | 0 to 15 minutes [ref] |  | 1 [ref] | 1 [ref] |
|  | 16 to 30 minutes |  | 2.13 *** [1.86-2.45] | 1.7 *** [1.49-1.95] |
|  | 31 to 60 minutes |  | 3.47 *** [2.92-4.11] | 2.74 *** [2.29-3.27] |
|  | Over 1 hour |  | 4.46 *** [3.12-6.38] | 3.16 *** [2.29-4.37] |
| Transport to health facility | Motorized travel to facility [ref] |  | 1 [ref] | 1 [ref] |
|  | Non-motorized travel to facility |  | 0.91 [0.79-1.05] | 0.81 *** [0.71-0.93] |
| Province | Koshi [ref] |  |  | 1 [ref] |
|  | Madhesh |  |  | 1.15 [0.92-1.44] |
|  | Bagmati |  |  | 0.75 ** [0.6-0.94] |
|  | Gandaki |  |  | 0.84 [0.64-1.09] |
|  | Lumbini |  |  | 0.71 *** [0.56-0.89] |
|  | Karnali |  |  | 1.25 * [0.98-1.58] |
|  | Sudurpashchim |  |  | 0.52 *** [0.42-0.65] |
| Residence | Urban [ref] |  |  | 1 [ref] |
|  | Rural |  |  | 0.94 [0.83-1.08] |
| Caste | Hill Brahmin/Chhetri [ref] |  |  | 1 [ref] |
|  | Terai/Madheshi |  |  | 1.4 *** [1.14-1.71] |
|  | Dalit |  |  | 1.18 ** [1.01-1.39] |
|  | Janajatis |  |  | 1.26 *** [1.1-1.44] |
|  | Muslim/others |  |  | 1.6 *** [1.21-2.11] |
| Marital status | In union [ref] |  |  | 1 [ref] |
|  | Never In union |  |  | 1.03 [0.89-1.19] |
|  | Formerly In union |  |  | 0.99 [0.79-1.23] |
| Education | None |  |  | 1.2 *** [1.06-1.35] |
|  | Basic [ref] |  |  | 1 [ref] |
|  | Secondary |  |  | 0.7 *** [0.63-0.78] |
|  | Higher |  |  | 0.51 *** [0.4-0.65] |
| Wealth | Poorest |  |  | 1.33 *** [1.14-1.56] |
|  | Poorer |  |  | 1.1 [0.97-1.24] |
|  | Middle [ref] |  |  | 1 [ref] |
|  | Richer |  |  | 0.81 *** [0.7-0.92] |
|  | Richest |  |  | 0.76 *** [0.63-0.9] |
| De jure household size | 1 member |  |  | 0.67 ** [0.49-0.91] |
|  | 2 members |  |  | 0.89 [0.76-1.05] |
|  | 3 members |  |  | 0.93 [0.82-1.06] |
|  | 4 members [ref] |  |  | 1 [ref] |
|  | 5 members |  |  | 0.93 [0.82-1.05] |
|  | 6 members |  |  | 0.97 [0.84-1.13] |
|  | 7+ members |  |  | 1.05 [0.9-1.22] |
| Age | 15–19 |  |  | 1.7 *** [1.39-2.07] |
|  | 20–24 |  |  | 1.34 *** [1.16-1.56] |
|  | 25–29 |  |  | 1.08 [0.92-1.26] |
|  | 30–34 [ref] |  |  | 1 [ref] |
|  | 35–39 |  |  | 1.01 [0.87-1.18] |
|  | 40–44 |  |  | 1.12 [0.96-1.32] |
|  | 45–49 |  |  | 1.16 * [0.97-1.38] |
| Self rates health | Very good |  |  | 0.53 *** [0.41-0.69] |
|  | Good [ref] |  |  | 1 [ref] |
|  | Moderate |  |  | 1.23 *** [1.09-1.38] |
|  | Bad/very bad |  |  | 1.45 *** [1.21-1.73] |
| Occupation | Employed [ref] |  |  | 1 [ref] |
|  | Agriculture |  |  | 1.3 *** [1.13-1.5] |
|  | Manual |  |  | 1.22 ** [1.01-1.47] |
|  | Unemployed/others |  |  | 1.38 *** [1.19-1.6] |
|  | | | | |
| Note: *** = Significant at p<0.001; **=p<0.01, and *=p<0.05 | | | | |
|  | | | | |
